# Foodborne Botulism Presenting With Isolated Cranial Nerve Involvement in a Young Adult

**DOI:** 10.7759/cureus.97040

**Published:** 2025-11-17

**Authors:** Miguel Barbosa, Maria E Batista, Daniela Carneiro, Sofia Cardoso, Nuno Germano

**Affiliations:** 1 Department of Critical Care Medicine, Hospital Curry Cabral, Unidade Local de Saúde de São José, Lisbon, PRT

**Keywords:** bulbar symptoms, cranial nerve paralysis, diagnosis of rare cases, foodborne botulism, medical intensive care unit (micu)

## Abstract

A young man presented with progressive cranial nerve dysfunction following participation in a communal meal. Initial investigations and imaging were inconclusive; Guillain-Barré syndrome and viral encephalitis were considered in the differential diagnosis. He was admitted to the intensive care unit (ICU) due to the potential need for respiratory support. The progression of neurological dysfunction, combined with relevant dietary exposure, raised clinical suspicion for botulism. The patient was treated with heptavalent botulinum antitoxin, and serum testing later confirmed the presence of botulinum toxin type A. With multidisciplinary supportive care, he fully recovered without requiring mechanical ventilation. This case highlights the importance of early recognition of bulbar symptoms, the timely administration of empirical antitoxin therapy, and the role of ICU monitoring. It also underscores how non-specific early symptoms can delay diagnosis and emphasizes the need for high clinical suspicion in otherwise healthy young patients.

## Introduction

Botulism is a rare neurological disorder caused by botulinum neurotoxins, produced primarily by *Clostridium botulinum* and related species [1]. There are seven serotypes of botulinum toxins (from A to G), with types A, B, and E being the most frequently associated with human disease. Botulism can occur in several clinical forms: foodborne (via ingestion of preformed toxin), wound (toxin production in infected tissue), infant (intestinal colonization), and adult intestinal toxemia [2]. The route of exposure also influences the incubation period, with foodborne botulism typically having the shortest latency. In Europe, where the notification of botulism is compulsory, 84 cases were reported in 2022, including one in Portugal, mostly caused by type B (85%) neurotoxin [3]. Clinical manifestations can vary depending on the route of exposure. In adults, foodborne botulism typically presents with an acute onset of cranial nerve palsies, such as diplopia, ptosis, dysarthria, and dysphagia, followed by symmetrical descending flaccid paralysis. Pupillary dilation and signs of autonomic dysfunction may also occur. Due to its rarity and non-specific early symptoms, botulism can easily be mistaken for conditions like Guillain-Barré syndrome (GBS), myasthenia gravis, or brainstem stroke [4]. While laboratory confirmation can take several days, botulism remains a clinical diagnosis, and prompt administration of antitoxin is critical to prevent progression. Given its public health implications, botulism is a notifiable disease in Europe and worldwide.

## Case presentation

History of presenting illness

A patient in his late teens with no prior medical history presented to the emergency department with a three-day history of progressive cranial nerve dysfunction. The initial symptom was a change in voice quality, described as nasal. Within 24 hours, he developed perioral weakness, difficulty articulating speech, and nasal regurgitation during meals. He subsequently experienced difficulty in closing his eyes and noted a loss of facial expressiveness, which prompted him to seek urgent medical attention. He denied dyspnea, limb weakness, paresthesia, or vision changes. There were no constitutional symptoms such as fever, weight loss, or night sweats.

Physical examination

The patient was alert, calm, and fully oriented. Cranial nerve examination revealed marked dysphonia, bilateral facial paresis (more pronounced in the lower face), and dysphagia to both solids and liquids. He was able to protrude the tongue in the midline, though mild weakness was noted, and the gag reflex was preserved. Mild dysmetria was observed in the left upper limb on finger-to-nose testing. The pupils were 5 mm in diameter, symmetrical, and non-reactive to light. There was no ptosis or ophthalmoparesis. Motor and sensory examinations of the limbs were normal, and deep tendon reflexes were preserved and symmetrical. There were no signs of meningeal irritation.

Cardiovascular, respiratory, and abdominal examinations were unremarkable. He remained afebrile and hemodynamically stable throughout.

Epidemiological and occupational exposure

The patient was a student who had participated in a university tradition in the preceding week. This involved prolonged vocal exertion, physical activities, and communal meals under suboptimal hygienic conditions. During this period, he consumed canned food of uncertain origin with an unusual taste. He also reported a single episode of non-bloody diarrhea shortly after consumption, but no further gastrointestinal symptoms.

In the same last three days of this activity, he developed an odynophagia and voice alteration. He self-treated with milk tea containing homemade honey, obtained from a family acquaintance. He could not confirm whether others had consumed the same honey, but no one else he knew experienced similar symptoms.

Social and environmental history

He resided in an urban household with his parents and sibling. He denied recent travel, exposure to animals, insect bites, or recreational drug use. No close contacts reported similar symptoms. There was no known family or community cluster of neurological or gastrointestinal illness. Given the risk of respiratory compromise, the patient was admitted to the intensive care unit (ICU) for close monitoring and further diagnostic evaluation.

Investigation

Initial biochemical and hematological analyses, including inflammatory markers, renal and liver function tests, and complete blood count, were within normal limits. A computed tomography (CT) scan of the brain revealed no acute pathology.

Lumbar puncture was performed, normal opening pressure was observed, and cerebrospinal fluid (CSF) analysis showed 3 leukocytes/μL, glucose of 62 mg/dL, and protein concentration of 21 mg/dL. There was no albuminocytologic dissociation. Direct microbiological examination was negative. CSF was sent for viral polymerase chain reaction (PCR) panel and bacterial cultures, both of which were negative.

Given the clinical presentation, GBS, particularly the Miller Fisher variant, was initially suspected. Magnetic resonance imaging (MRI) of the brain and cervical spine was performed and revealed no abnormalities. Autoimmune serology, including antinuclear antibodies and ganglioside antibodies (e.g., anti-GQ1b), was negative. Acetylcholine receptor and muscle-specific kinase antibodies were also absent.

Viral encephalitis was considered; however, a broad neurotropic viral PCR panel from CSF was negative, effectively excluding common viral etiologies. Due to the predominance of cranial nerve involvement and the epidemiological context, botulism was suspected. Serum and stool samples were submitted for the detection of botulinum neurotoxin using enzyme-linked immunosorbent assay (ELISA). Botulinum toxin type A was identified in the serum sample, while stool analysis was negative. A confirmatory mouse lethality bioassay (MLB), considered the diagnostic gold standard, was performed but returned negative.

Differential Diagnosis

Given the patient’s presentation with progressive cranial nerve dysfunction, dysphagia, dysphonia, and bilateral facial weakness, three main differential diagnoses were considered. A summary of the comparison is illustrated in Table 1.

**Table 1:** Comparison between botulism, Guillain-Barré syndrome, and viral encephalitis. This table compares key clinical features of botulism, Guillain-Barré syndrome, and viral encephalitis. It highlights differences in onset pattern, cranial nerve involvement, and pupillary responses, which are useful in differentiating these neurologic conditions.

Features	Botulism	Guillain-Barré syndrome	Viral encephalitis
Onset/incubation	Foodborne: 12–72 h	Days to weeks post-infection	1–7 days after viral prodrome
Initial symptoms	Cranial nerve palsies	Limb weakness, paresthesia	Fever, headache, altered mental status
Weakness pattern	Descending, symmetrical	Ascending symmetrical paralysis	Variable: focal deficits possible
Sensory findings	Usually spared	Mild sensory loss	Often preserved or variable
Reflexes	Normal or decreased late	Absent or decreased	Usually preserved
Cranial nerves	Early involvement	Sometimes involved	Variable, depending on the brain region affected
Mental status	Preserved	Preserved	Altered
Pupils	Dilated, non-reactive	Rarely affected	Variable, depending on severity

GBS, particularly the Miller Fisher variant, was initially favored due to its higher relative frequency in developed countries and its potential post-infectious etiology. The patient reported a recent upper respiratory tract infection, which could have acted as a triggering event. Although classical features such as ophthalmoplegia, areflexia, and ataxia were absent or only subtly present, the early and atypical clinical presentation, coupled with the potential for rapid progression, kept GBS within the diagnostic considerations. However, the absence of albuminocytologic dissociation in the CSF, negative antiganglioside antibodies, and unremarkable neuroimaging made this diagnosis less likely.

Viral encephalitis was also considered. However, the patient exhibited no alteration in consciousness, cognitive impairment, or fever. MRI was unremarkable, and CSF findings were non-inflammatory. These factors, along with a negative viral PCR panel, argued strongly against this diagnosis.

Botulism emerged as a leading differential due to the isolated and progressive cranial nerve involvement without accompanying fever, sensory changes, or limb weakness. The pattern of cranial neuropathies was notable: involvement of facial muscles, bulbar dysfunction affecting mastication and swallowing, and ocular involvement limited to eyelid muscles without ophthalmoplegia. The pupillary alteration also supported this diagnosis.

**Figure 1:**
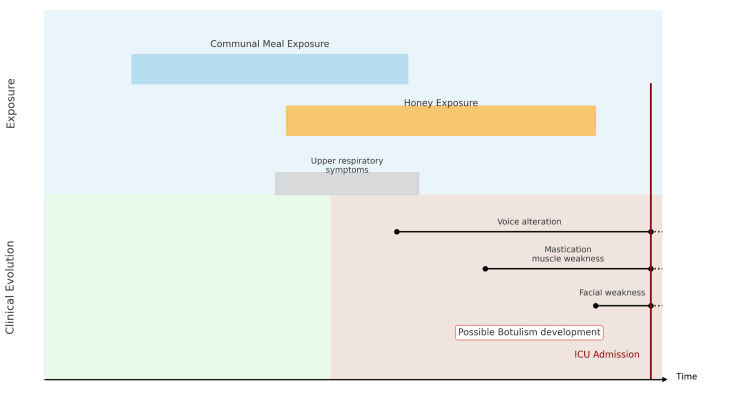
Exposure and clinical evolution landmarks until ICU admission. The timeline illustrates key exposures, symptom onset, and clinical deterioration. The orange-shaded area represents the period of uncertainty regarding the progression of botulism. The absence of other reported cases and the unclear origin of toxin exposure made the diagnosis particularly challenging. ICU: intensive care unit

Epidemiologically, the patient reported ingestion of canned food with an abnormal taste and consumption of homemade honey. Figure 1 presents the relationship between symptoms and possible exposure. While honey is classically associated with infant botulism, its implication in adult cases is rare but not impossible. Despite the absence of a confirmed outbreak or known co-exposed individuals, the timing of exposure and clinical presentation warranted testing, which ultimately confirmed the presence of botulinum toxin type A in serum by ELISA. The MLB was negative; however, both ELISA and bioassay sensitivity can vary depending on specimen quality, timing of collection, and toxin concentration, with ELISA being more rapid but less sensitive at low toxin levels, and the bioassay potentially affected by sample degradation or delay in processing.

Treatment

The patient was admitted to the ICU for close respiratory and neurological monitoring due to the risk of respiratory failure associated with progressive cranial nerve involvement. On admission, he exhibited oropharyngeal dysphagia, impaired lingual mobility, and bilateral facial weakness. Despite these findings, his respiratory mechanics remained stable, with no need for supplemental oxygen or ventilatory support.

A multidisciplinary evaluation was initiated, including consultations with physiotherapy and speech and language therapy services. Due to the high risk of aspiration, a nasogastric tube was placed to ensure adequate nutritional support.

Empirical treatment with intravenous human immunoglobulin (IVIG) was initiated at a dose of 0.4 g/kg/day (24 g daily for an estimated 60 kg body weight). Although albuminocytologic dissociation is a classic finding in GBS, its sensitivity depends on the timing of lumbar puncture. It has been reported to range between 44% and 81%, with lower sensitivity early in the disease course [4]. The patient completed three days of IVIG without clinical improvement, which prompted reconsideration of the initial diagnosis.

Following the possible botulism diagnosis, the patient received a single dose of heptavalent botulinum antitoxin (HBAT) as per national public health protocol. Remarkably, he reported subjective improvement in lingual motor function shortly after the antitoxin infusion, noting improved tongue mobility.

In the days following antitoxin administration, the patient continued to show gradual clinical improvement. He received daily physiotherapy focused on maintaining functional mobility and preventing deconditioning. Weekly speech and swallowing therapy sessions were also conducted to improve buccal and pharyngeal motor control. He remained in the ICU for one week for observation and supportive care and was subsequently transferred to a rehabilitation ward to continue recovery and functional reconditioning.

Outcome and follow-up

The patient completed a total of 45 days of inpatient care, including intensive care monitoring and a multidisciplinary rehabilitation program. Over the course of his admission, he showed steady neurological improvement, with resolution of dysphagia, recovery of facial motor function, and restoration of normal speech.

Upon discharge, he no longer required nasogastric feeding, had resumed an unrestricted oral diet, and was independently mobile. At follow-up in the outpatient neurology consultation, his neurological examination was entirely normal, with no residual cranial nerve deficits or limb weakness.

## Discussion

Botulism is a rare but life-threatening syndrome caused by botulinum neurotoxins, which irreversibly block acetylcholine release at peripheral cholinergic synapses. In foodborne botulism, the most common form in Europe, the disease follows ingestion of preformed neurotoxin typically from improperly preserved or contaminated food [5]. The case presented here exemplifies key diagnostic and therapeutic challenges, particularly in early recognition, differential diagnosis, and the timing of antitoxin therapy.

Botulinum neurotoxins, particularly types A, B, and E (which are most relevant to human disease), act at the presynaptic terminal of neuromuscular junctions by cleaving SNARE proteins essential for acetylcholine release [6]. This results in the hallmark flaccid descending paralysis, classically starting with cranial nerves and progressing caudally. In this case, the patient developed early cranial nerve dysfunction (dysphonia, bilateral facial weakness, and dysphagia) without initial limb weakness or respiratory compromise. This is consistent with the early phase of type A intoxication, which is known for more severe and prolonged symptoms compared to types B or E [7].

Due to the rarity of botulism and its overlapping features with other neuromuscular disorders, misdiagnosis is common [8]. GBS, particularly its Miller Fisher variant, is a common initial consideration due to overlapping signs such as ophthalmoparesis, ataxia, and facial diplegia. However, preserved reflexes, lack of sensory symptoms, normal CSF, and absence of antiganglioside antibodies (as in our case) argue against GBS. Similarly, the absence of fatigable weakness and acetylcholine receptor antibodies made myasthenia gravis unlikely [7].

The Centers for Disease Control and Prevention (CDC) recommends initiating antitoxin therapy based on clinical suspicion rather than waiting for laboratory confirmation [3]. Diagnostic delay is associated with worse outcomes, including respiratory failure and prolonged hospitalization. In our case, ELISA confirmed BoNT type A in serum, but stool and mouse bioassays were negative, which may reflect sample timing or sensitivity limitations. Notably, positive ELISA results in serum alone are sufficient for confirmation under most public health criteria [9]. Recent CDC-supported criteria suggest that afebrile patients presenting with ≥2 of the following-dysphagia, ptosis, diplopia, or descending weakness should be urgently evaluated for botulism, especially if gastrointestinal or dietary history supports exposure risk [3,10].

According to WHO and CDC guidelines, HBAT should be administered as early as possible to neutralize circulating toxin. The antitoxin does not reverse established paralysis but may prevent further progression [3,7]. Our patient demonstrated subjective improvement in tongue movement shortly after infusion, consistent with prior studies reporting early symptom relief even after several days of illness [11]. Supportive care-including airway protection, nutritional support, and early rehabilitation-is central to management and long-term recovery.

This case highlights a rare but essential diagnosis that may be missed due to its low prevalence and non-specific early symptoms. It also underscores the need for clinical vigilance, especially when caring for young, otherwise healthy adults with isolated cranial neuropathies and a history of dietary exposures. Though adult botulism from honey is rare, it cannot be entirely ruled out, particularly in individuals with altered gut microbiota or subclinical mucosal injuries [12]. The ingestion of suspect canned food further supports the hypothesis of classic foodborne transmission.

## Conclusions

This case illustrates how the subtle and non-specific early symptoms of botulism can contribute to diagnostic delay. In this instance, the absence of limb involvement initially complicated recognition. While objective measures of respiratory and swallowing function were limited, the clinical evolution warranted ICU admission for close monitoring, reflecting the real risk of sudden diaphragmatic or upper airway paralysis in botulism.

This case emphasizes the importance of maintaining botulism in the differential diagnosis of descending cranial neuropathies, even in the absence of classical risk factors. A systematic neurological assessment and early involvement of critical care and multidisciplinary teams were essential in the patient’s recovery.
